# Supplementary material for: Oxytocin promotes coordinated out-group attack during intergroup conflict in humans
Source: eLife. 2019 Jan 25;8:e40698. doi: 10.7554/eLife.40698 (PMC6347450; doi:10.7554/eLife.40698)
Supplement: Supplementary file 1. — (A) Table 1A. Match demographic information and prosocial-related traits. (B) Table 1B. Mood changes from pre-experiment to post-experiment. (C) Table 1C. Point estimates for indirect effects and bootstrapped 95% bias-corrected confidence intervals for multiple mediational analysis in which attacker group’s tracking (strategic tracking when α→ −1) and within-group variance (variance) were represented as mediators in the association between Treatment and spoils from winning a conflict during simultaneous decision-making. (D) Table 1D. Point estimates for indirect effects and bootstrapped 95% bias-corrected confidence intervals for multiple mediational analysis in which attacker group’s tracking (strategic tracking when α→ −1) and within-group variance (variance) were represented as mediators in the association between Treatment and spoils and leftovers during simultaneous decision-making. [file elife-40698-supp1.docx]

**Supplementary file 1**

**Supplementary Tables 1A through 1D**

**Supplementary file 1. Table 1A.** Match demographic information and prosocial-related traits.

|  | **Oxytocin** | | **Placebo** | | **Role** | **Treatment** | **Interaction** |
| --- | --- | --- | --- | --- | --- | --- | --- |
|  | **Attackers** | **Defenders** | **Attackers** | **Defenders** |  |  |  |
|  | Mean (SE) | Mean (SE) | Mean (SE) | Mean (SE) | *p* | *p* | *p* |
| Age (year) | 20.25 (0.16) | 20.21 (0.18) | 20.43 (0.13) | 20.25 (0.16) | 0.159 | 0.889 | 0.290 |
| Education (year) | 15.82 (0.09) | 15.85 (0.07) | 15.71 (0.10) | 15.86 (0.06) | 0.249 | 0.646 | 0.468 |
| Empathic capacity | 70.00 (0.91) | 69.35 (1.03) | 70.29 (1.18) | 70.16 (0.99) | 0.697 | 0.602 | 0.795 |
| Cooperative personality | 48.53 (0.43) | 49.05 (0.43) | 49.64 (0.42) | 49.98 (0.51) | 0.502 | 0.113 | 0.852 |
| Social value orientation | 27.14 (1.15) | 27.73 (1.05) | 25.99 (1.15) | 26.23 (1.18) | 0.703 | 0.268 | 0.876 |
| Prosocial personality | 41.19 (0.50) | 41.51 (0.41) | 40.96 (0.51) | 40.86 (0.48) | 0.809 | 0.401 | 0.625 |
| Impulsiveness | 39.97 (0.46) | 39.50 (0.42) | 40.14 (0.43) | 40.50 (0.47) | 0.902 | 0.217 | 0.332 |
| Socio-economic status | 5.24 (0.12) | 5.31 (0.16) | 5.49 (0.15) | 5.09 (0.13) | 0.217 | 0.934 | 0.090 |

Table note: We conducted 2 (Treatment: placebo vs. oxytocin) × 2 (Role: attacker vs. defender) ANOVAs on the demographic information and prosocial-related traits. Empathic capacity was measured using the Interpersonal Reactivity Index (Davis, 1983), which consists of 28 items on a 5-point (0-4) Likert scale. Cooperative personality was measured by the cooperative subscale of the Cooperation and Competition Personality scale (Xie et al., 2006; 13 items on a 5-point (1-5) Likert scale). Social value orientation was measured by the 6 primary items of the Social Value Orientation Slider task (Murphy et al., 2011). Prosocial personality was measured by the Social Responsibility, Other-Oriented Moral Reasoning and Mutual Concerns Moral Reasoning subscales of Prosocial Personality Battery (Penner, 2002). Impulsiveness was measured using BAS subscale of the Behavioral Inhibition/Behavioral Activation Scales, BIS/BAS (Carver and White, 1994), which consists of 13 items on 4-point (1-4) Likert scale. Subjective socio-economic status was measured by a typical Subjective Socioeconomic Status Ladder Scale (Operario et al., 2004) with 10 rungs with the instruction of “Imagine that this ladder with 10 rungs represents where people stand in our society. At the top are the people who are best off – those who have the most money, highest education, and best jobs. At the bottom are the people who are the worst off – those who have the least money, lowest education, and the worst jobs or no job. Please mark the rung that best represents where you think you stand in the society.”

Supplementary file 1. Table 1B. Mood changes from pre-experiment to post-experiment.

|  | | **Oxytocin** | | | **Placebo** | | **Main effect of Role** | **Main effect of Treatment** | **Role x Treatment**  **Interaction** |
| --- | --- | --- | --- | --- | --- | --- | --- | --- | --- |
|  | Attacker | | Defender | Attacker | | Defender |  |  |  |
|  | Mean (SE) | | Mean (SE) | Mean (SE) | | Mean (SE) | *p* | *p* | *p* |
| **Positive** | 0.00 (0.12) | | 0.04 (0.11) | 0.02 (0.09) | | 0.18 (0.10) | 0.357 | 0.457 | 0.620 |
| **Negative** | -0.01(0.12) | | -0.17 (0.13) | -0.01 (0.12) | | -0.15 (0.12) | 0.202 | 0.930 | 0.937 |
| **Overall** | -0.01 (0.08) | | -0.06 (0.07) | 0.01 (0.06) | | 0.02 (0.07) | 0.764 | 0.525 | 0.649 |

Table note: We conducted 2 (Treatment: placebo vs. oxytocin) × 2 (Role: attacker vs. defender) ANOVAs on the mood change of the positive, negative and overall mood respectively.

Supplementary file 1. Table 1C. Point estimates for indirect effects and bootstrapped 95% bias-corrected confidence intervals for multiple mediational analysis in which attacker group’s tracking (*strategic tracking when α* 🡪 *-1*) and within-group variance (*variance*) were represented as mediators in the association between *Treatment* and *spoils from winning* a conflict during simultaneous decision-making.

| **Path** | Estimate | SE | LLCI | ULCI |
| --- | --- | --- | --- | --- |
| Treatment 🡪 Attackers track defenders (α) 🡪 Spoils from winning | 0.165 | 0.342 | -0.395 | 0.945 |
| Treatment 🡪 Variance 🡪 Spoils from winning | 0.047 | 0.197 | -0.321 | 0.509 |
| **Treatment** 🡪 **Attackers track defenders (α)** 🡪 **Variance** 🡪 **Spoils from winning** | **0.147** | **0.103** | **0.021** | **0.510** |
| Total indirect effect | 0.359 | 0.360 | -0.168 | 1.280 |

Table note: Treatment was a dichotomous variable (0 = placebo; 1 = oxytocin); Confidence intervals for indirect effect are bias-corrected and accelerated bootstrap resamples=5000; *N=76* for all tests.

Supplementary file 1. Table 1D. Point estimates for indirect effects and bootstrapped 95% bias-corrected confidence intervals for multiple mediational analysis in which attacker group’s tracking (*strategic tracking when α* 🡪 *-1)* and within-group variance (*variance*) were represented as mediators in the association between *Treatment* and *spoils and leftovers* during simultaneous decision-making.

| **Path** | Estimate | SE | LLCI | ULCI |
| --- | --- | --- | --- | --- |
| Treatment **🡪** Attackers track defenders (α) **🡪** Spoils & leftovers | 0.510 | 0.542 | -0.232 | 1.942 |
| Treatment **🡪** Variance **🡪** Spoils and leftovers | 0.069 | 0.303 | -0.488 | 0.738 |
| **Treatment 🡪 Attackers track defenders (α) 🡪 Variance 🡪 Spoils and leftovers** | **0.218** | **0.174** | **0.022** | **0.779** |
| Total indirect effect | 0.797 | 0.568 | -0.027 | 2.234 |

Table note: Treatment was a dichotomous variable (0 = placebo; 1 = oxytocin); Confidence intervals for indirect effect are bias-corrected and accelerated bootstrap resamples = 5000; *N=76* for all tests.

***Supplementary Reference***

Carver CS, White TL. 1994. Behavioral inhibition, behavioral activation, and affective responses to impending reward and punishment: the BIS/BAS scales. *Journal of Personality and Social Psychology* **67**: 319.

Davis MH. 1983. Measuring individual differences in empathy: Evidence for a multidimensional approach. *Journal of Personality and Social Psychology***44**: 113.

Murphy RO, Ackermann KA, Handgraaf M. 2011. Measuring social value orientation. *Judgment and Decision Making* **6**: 771-781.

Operario D, Adler NE, Williams DR. 2004. Subjective social status: Reliability and predictive utility for global health. *Psychology and Health* **19**: 237-246.

Penner LA. 2002. Dispositional and organizational influences on sustained volunteerism: An interactionist perspective. *Journal of Social Issues* ***58***: 447-467.

Xie X, Yu Y, Chen X, Chen X. 2006. The Measurement of Cooperative and Competitive Personality. *Acta Psychologica Sinica* **38**: 116-125.
