# Supplementary material for: Oxytocin promotes coordinated out-group attack during intergroup conflict in humans
Source: eLife. 2019 Jan 25;8:e40698. doi: 10.7554/eLife.40698 (PMC6347450; doi:10.7554/eLife.40698)
Supplement: Transparent reporting form [file elife-40698-transrepform.docx]

***eLife’s* transparent reporting form**

We encourage authors to provide detailed information *within their submission* to facilitate the interpretation and replication of experiments. Authors can upload supporting documentation to indicate the use of appropriate reporting guidelines for health-related research (see [EQUATOR Network](http://www.equator-network.org/%20)), life science research (see the [BioSharing Information Resource](https://biosharing.org/)), or the [ARRIVE guidelines](http://www.plosbiology.org/article/info:doi/10.1371/journal.pbio.1000412) for reporting work involving animal research. Where applicable, authors should refer to any relevant reporting standards documents in this form.

If you have any questions, please consult our Journal Policies and/or contact us: [editorial@elifesciences.org](mailto:editorial@elifesciences.org).

**Sample-size estimation**

- You should state whether an appropriate sample size was computed when the study was being designed
- You should state the statistical method of sample size computation and any required assumptions
- If no explicit power analysis was used, you should describe how you decided what sample (replicate) size (number) to use

Please outline where this information can be found within the submission (e.g., sections or figure legends), or explain why this information doesn’t apply to your submission:

The data analysis on the current study was conducted on a 6-person-group level, thus we conducted sample size estimation by G*Power to determine the number of groups sufficient to detect a reliable effect. Based on an estimated average small-to-medium effect size of oxytocin effect on social behaviors (d = 0.28, Walum et al., 2016), 80 6-person groups were needed to detect a significant effect (α = 0.05, β = 0.85, ANOVA: repeated measures, within-between interaction, G-Power, Faul et al., 2009).

This is mentioned in the Participants section under Materials and Methods.

**Replicates**

- You should report how often each experiment was performed
- You should include a definition of biological versus technical replication
- The data obtained should be provided and sufficient information should be provided to indicate the number of independent biological and/or technical replicates
- If you encountered any outliers, you should describe how these were handled
- Criteria for exclusion/inclusion of data should be clearly stated
- High-throughput sequence data should be uploaded before submission, with a private link for reviewers provided (these are available from both GEO and ArrayExpress)

Please outline where this information can be found within the submission (e.g., sections or figure legends), or explain why this information doesn’t apply to your submission:

The experiment was performed once.

The oxytocin effect on increasing within-group coordination was observed when measuring within-group variance (Figure 2A), as well as calculating the intra-class correlation coefficient (Figure S4). The relationship that better coordination predicted payoff for attacker group was observed when only considering the spoils (Figure 3B-D), as well as considering both spoils and leftover (Figure S6).

Criteria for inclusion of participants are stated in the Participants section under Materials and Methods.

**Statistical reporting**

- Statistical analysis methods should be described and justified
- Raw data should be presented in figures whenever informative to do so (typically when N per group is less than 10)
- For each experiment, you should identify the statistical tests used, exact values of N, definitions of center, methods of multiple test correction, and dispersion and precision measures (e.g., mean, median, SD, SEM, confidence intervals; and, for the major substantive results, a measure of effect size (e.g., Pearson's r, Cohen's d)
- Report exact p-values wherever possible alongside the summary statistics and 95% confidence intervals. These should be reported for all key questions and not only when the p-value is less than 0.05.

Please outline where this information can be found within the submission (e.g., sections or figure legends), or explain why this information doesn’t apply to your submission:

Details for the statistical methods are reported in the Materials and Methods sections.

Where appropriate, Mean, SEM, F/t, r values and effect size are reported in the Results section.

(For large datasets, or papers with a very large number of statistical tests, you may upload a single table file with tests, Ns, etc., with reference to sections in the manuscript.)

**Group allocation**

- Indicate how samples were allocated into experimental groups (in the case of clinical studies, please specify allocation to treatment method); if randomization was used, please also state if restricted randomization was applied
- Indicate if masking was used during group allocation, data collection and/or data analysis

Please outline where this information can be found within the submission (e.g., sections or figure legends), or explain why this information doesn’t apply to your submission:

The experiment followed a randomized double-blind placebo-controlled between-subjects design. The 6-person group was randomly assigned to one of the two treatment conditions (oxytocin vs. placebo). The 6 individuals in each IADC session randomly assigned to a 3-person attacker and a 3-person defender group.

The information can be found in the Methods Summary section, and the Procedure section under Materials and Methods.

**Additional data files (“source data”)**

- We encourage you to upload relevant additional data files, such as numerical data that are represented as a graph in a figure, or as a summary table
- Where provided, these should be in the most useful format, and they can be uploaded as “Source data” files linked to a main figure or table
- Include model definition files including the full list of parameters used
- Include code used for data analysis (e.g., R, MatLab)
- Avoid stating that data files are “available upon request”

Please indicate the figures or tables for which source data files have been provided:

The group-level statistics are plotted in Figures 1-3, Figures S3-S6. The individual data is plotted in Figures 3 and S6. The source data for Figures 1-3 is publicly available at https://osf.io/7jeas/.
